# Seed ecology of the geophyte *Conopodium majus* (Apiaceae), indicator species of ancient woodland understories and oligotrophic meadows

**DOI:** 10.1111/plb.12872

**Published:** 2018-08-08

**Authors:** C. Blandino, E. Fernández‐Pascual, M. Marin, A. Vernet, H. W. Pritchard

**Affiliations:** ^1^ Comparative Plant and Fungal Biology Royal Botanic Gardens Kew West Sussex UK; ^2^ Earth and Environmental Sciences Department University of Pavia Pavia Italy; ^3^ Departamento de Biología de Organismos y Sistemas Universidad de Oviedo Oviedo/Uviéu Spain; ^4^ Scotia Seeds Brechin UK; ^5^ Division of Cardiovascular Medicine Wellcome Trust Centre For Human Genetics Radcliffe UK

**Keywords:** Desiccation tolerance, embryo morphology, morphological dormancy, pseudomonocotyly, seed germination, seed storage

## Abstract

- *Conopodium majus* is a geophyte with pseudomonocotyly, distributed in Atlantic Europe. It is an indicator of two declining European habitats: ancient woodland understories and oligotrophic hay meadows. Attempts to reintroduce it by seed have been hindered by scarce seedling emergence and limited knowledge of its seed biology.
- Micro‐CT scanning was used to assess pseudomonocotyly. Embryo growth and germination were studied in the laboratory and the field, using dissection and image analysis. The effects of temperature, light, nitrate and GA_3_ on germination were tested. Seed desiccation tolerance was investigated by storage at different RHs and by drying seeds at different stages of embryo growth.
- Seeds possess morphological but not physiological dormancy. Embryo growth and germination were promoted by temperatures between 0 and 5 °C, arrested above 10 °C, and indifferent to alternating temperatures, light, nitrate and GA_3_. Pseudomonocotyly appears to result from cotyledon fusion. While seeds tolerated drying to 15% RH and storage for 1 year at 20 °C, viability was lost when storage was at 60% RH. Seeds imbibed at 5 °C for 84 days had significant internal embryo growth but were still able to tolerate drying to 15% RH.
- Reproduction by seed in *C. majus* follows a strategy shared by geophytes adapted to deciduous temperate forests. The evolution of fused cotyledons may enable the radicle and the hypocotyl to reach deeper into the soil where a tuber can develop. The embryo is capable of growth within the seed at low temperatures so that germination is timed for early spring.

## Introduction

Temperate deciduous forests are characterized by a predictable seasonality. During the growing season, the tree canopy closes and limits the amount of light that reaches the understorey. Leaf senescence in autumn opens the canopy and increases light availability for the understorey plants, but at the same time cold temperatures can become limiting for growth (Archibold 1995). In Europe, the majority of understorey herbaceous plants possess dormant seeds, allowing alignment between early life‐cycle events and forest seasonality (Baskin & Baskin 2014). Often temperate forest species produce seeds with morphological dormancy (MD). In MD seeds, the embryo is small, undifferentiated or underdeveloped at the time of seed dispersal, and needs to grow within the seed before germination can occur (Baskin & Baskin 2004). Embryo growth within the seed can delay germination until the end of a long and predictable unfavourable season, such as the period of canopy closure. For this reason, embryo growth may require months of exposure to temperatures characteristic of that season. In some species with MD, embryo growth does not start until an additional physiological block is released, a trait known as morphophysiological dormancy (MPD), which has been suggested to be the ancestral character of all seed plants (Willis *et al*. 2014). MD is highly conserved within some plant lineages (Martin 1946; Finch‐Savage & Leubner‐Metzger 2006). For example, underdeveloped, linear embryos are a major feature of the *Apiaceae* (Martin 1946). MD occurs in 96% of *Apiaceae* species and most have a combined MPD (Willis *et al*. 2014). Embryo traits in this family have been related to habitat preferences, with species adapted to shady habitats having smaller embryos that require cold temperatures for growth (Vandelook *et al*. 2012).

The vernal geophyte *Conopodium majus* (Gouan) Loret presents an interesting system to study the germination ecology of the *Apiaceae*, due to its broad ecological niche throughout its sub‐Atlantic distribution in Europe. *C. majus* is considered an Ancient Woodland Indicator in both Britain (Kirby 2006) and continental Europe (Hermy *et al*. 1999). However, it is also a characteristic species of open hay meadows (Losvik 1993) on nutrient‐poor soils (Grime *et al*. 2007). Its establishment is inhibited by competing vegetation when introduced to productive meadows (Thompson & Baster 1992), and its distribution in open meadows could be an indication of past woodland presence in the site (Grime *et al*. 2007). At the southern edge of its distribution in the Sistema Central of Spain, the species is confined to continental Mediterranean mountain habitats, where it can colonise forests, scrublands, meadows or rocky grasslands (Mateo & Lòpez Udias 2003). *C. majus* regenerates mostly by seed and there is little evidence that the species reproduces vegetatively (Lovett Doust & Lovett Doust 1982). The seeds do not show morphological adaptations to a specific dispersal agent and evidence of autochory (*e.g*. explosive dispersal) is absent (Grime *et al*. 2007). Therefore, the seeds have a limited dispersal capacity that, together with their short persistence in the soil (Thompson *et al*. 1997), define *C. majus* as a poor coloniser of new habitats. Such poor colonising capacity of *C. majus,* a species that has been reported as declining at least in Britain (Grime *et al*. 2007), makes it a target plant for ecological restoration in both woodlands and oligotrophic meadows.

In spite of the ecological and restoration interest in *C. majus*, its seed ecophysiology is poorly understood, possibly because of its slow germination and low rates of seedling survival. Seedlings of *C. majus* possess a single cotyledon (Thompson 1988), a rare phenomenon known as pseudomonocotyly. This morphological condition has been described for a few plant families (Titova 2000) and several *Apiaceae* genera, where it appears to be a characteristic of tuberous geophytes (Haccius 1952; Degtjareva *et al*. 2009; Kljuykov *et al*. 2014). Despite the relative uniqueness of this trait, its ecological relevance is poorly understood.

Seeds of *C. majus* are dispersed at the end of summer, but seedlings are reported to emerge in late winter (Roberts 1979) so, as in many other *Apiaceae*, the species may need a period of cold stratification to allow embryo growth. To determine whether the species has MD or MPD, insight is needed on how embryo growth and germination respond to temperature and gibberellic acid (Baskin & Baskin 2014). As *C. majus* grows in oligotrophic soils (Grime *et al*. 2007), nitrate may be a cue for its dormancy loss or germination (Alboresi *et al*. 2005). In the continental Mediterranean mountains where the species can be found, drought events are common. Therefore, mechanisms to survive desiccation may be important both for freshly dispersed seeds and for seeds containing growing embryos. Describing the storage behaviour and the desiccation tolerance of *C. majus* seeds during different stages of embryo development would provide a better understanding of the ecological resilience of geophytes in drought‐affected areas. Moreover, resolving these questions will contribute to the use of *C. majus* by the growing European native seed industry (De Vitis *et al*. 2017). In this study, we investigated the seed ecology of *C. majus* with the aim of revealing the seed internal morphology, the environmental control of embryo growth and germination, and the storage behaviour and desiccation tolerance of the seeds at different stages of embryo growth.

## Material and methods

### Seed collections

Fresh seeds were collected on 28 August 2015 and 23 August 2016, at Dalreoch Farm (56°44′ 47″ N, 3°32′ 25″ W; Perthshire, Scotland, UK). The entire population was sampled haphazardly, taking ripe fruits (dry and brownish) from at least 50 individuals per collection. Fruits were kept at ambient temperature and humidity for 3 days and then sent to the Royal Botanic Gardens, Kew, Wakehurst Place (England, UK). For shipping, fruits were placed in sealed plastic bags to avoid desiccation. Seeds were cleaned, and experiments were started upon delivery. Seed mass (96 individual seeds), moisture content (ten individual seeds; 17 h, 103 °C; ISTA 2017) and initial viability were measured on arrival only for the 2015 seed lot. Seeds collected in 2016 were only used to assess the effect of light exclusion on embryo growth. All the other experiments were performed with seeds collected in 2015.

### Tetrazolium chloride (TZ) staining

Initial seed viability of the 2015 collection was tested using 1% aqueous solution of 2,3,5‐triphenyl tetrazolium chloride (TZ). Twenty seeds were rehydrated above water for 12 h at 20 °C, and then placed on 1% agar for 24 h to reach full hydration. A slice of the seed coat was removed from the dorsal surface of each seed, the seeds covered with the TZ solution and then incubated at 30 °C in the dark for 24 h. Afterwards, a longitudinal cut of the seeds was made, and the embryo located. Seeds were considered viable when both embryo and endosperm were uniformly stained red.

### Internal morphology

In order to produce a quality image of the fused cotyledon, a sample seed, from the 2015 lot, incubated on agar at 5 °C for 56 days to allow some embryo growth, was fixed in FAA (formaldehyde‐acetic acid‐alcohol) solution and sent to the University of Oxford Wellcome Trust Centre for Human Genetics to be scanned using a Micro Computed Tomography (Micro‐CT) scanner (SkyScan 1172; SkyScan, Kontich, Belgium). The sample was mounted on a pin and imaged in air at a nominal isotropic pixel size of 1.97 μm (voltage, 40 kV; current, 250 μA; 900 projections over 180°). Scan time was 2 h per sample. The images projections were reconstructed with the NRecon software (SkyScan 1172) using the Feldkamp algorithm.

### Embryo growth and germination laboratory experiments

#### Germination experiments

Laboratory germination experiments were conducted to determine the germination requirements of *C. majus*. Before starting a germination test, seeds were suspended in water overnight at 20 °C and sown the following day in 9‐cm diameter Petri dishes containing 1% agar‐water. In all the treatments, seeds received a 12‐h photoperiod. When fluctuating temperature regimes were applied, the dark phase corresponded with the cold phase (LMS Cooled incubators; LMS, Sevenoaks, UK). For all the germination experiments, four replicates of 25 seeds each were used, and the control treatment always consisted of fresh seeds incubated at 5 °C on agar with a 12 h/12 h photoperiod and without any pre‐treatment. Germination tests were scored weekly until no germinating seeds were observed for four consecutive weeks. Both radicle (1 mm protrusion, from here on defined as ‘germination’) and cotyledon emergence (*i.e*. release from the seed coat) were scored during the stratification, storage and desiccation tolerance tests. Non‐germinated seeds at the end of the experiment were dissected to assess their viability, considering that the initial viability for the 2015 seed lot was 90%. Non‐viable seeds (lacking a firm endosperm and embryo) were excluded from the calculation of final germination percentages.

#### Embryo growth measurements

Relative embryo size (E:E ratio: ‘embryo to endosperm ratio’) was measured in both seed lots on arrival and during embryo growth experiments described below. In all cases, reported measurements were made on ten viable seeds that had been stained with TZ as described above. Embryos were extracted from the seed, and both the embryo and the endosperm were photographed using a camera (Carl Zeiss Axiocam Colour) mounted on a Stemi SV 11 Microscope (Carl Zeiss, Welwyn Garden City, UK). Embryo and endosperm lengths were measured from the photographs using the software Axiovision 3.1.2.1 (Carl Zeiss).

For testing embryo growth at different temperatures, the effect of nitrate and GA_3,_ and the effect of desiccation at different stages of embryo development, ten Petri dishes were sown for each treatment, each containing 15 seeds. Every 14 days, a Petri dish was randomly selected from each treatment and the E:E of ten viable seeds, randomly selected, was measured as described above.

#### Effect of temperature

Pre‐screening showed that germination and embryo growth were inhibited at temperatures above 10 °C, so the temperatures tested were 0, 5 and 10 °C. The effect of temperature fluctuation was tested using an alternating day/night regime of 10/0 °C.

#### Effects of GA_3_ and KNO_3_

The effects of GA_3_ and KNO_3_ were tested at 5 °C, a temperature shown to be most effective for embryo growth and germination. GA_3_ (0.0645 mm) or KNO_3_ (3, 10 or 30 mm) were added to the agar‐water germination substrate. A total of 5 ml l^−1^ of the GA_3_ or KNO_3_ molar solutions were added to 450 ml melted agar as it cooled below 50 °C to achieve the final concentrations noted.

#### Effect of light

Only embryo growth was tested in the absence of light. Petri dishes were individually wrapped in aluminium foil and then put in a black plastic bag. Every 14 days, a bag was retrieved and embryo growth measured. The test was carried out in an incubator at 5 °C (constant temperature). Germination *per se* was not tested in this experiment.

#### Effect of cold stratification

The effect of cold stratification on seeds imbibed at 5 °C for different periods of time was investigated to test if they acquire the ability to germinate at warmer temperatures after a cold stratification treatment. Seeds were kept at 5 °C for 0, 6, 14 and 20 weeks, and then moved to 10 °C or 15/5 °C (12/12 h). The control treatment, in which seeds were held at 5 °C and not moved to warmer temperatures, was the same control used for testing the effect of nitrate, GA_3_ and storage conditions.

### Embryo growth in soil

To monitor embryo growth in natural conditions, 20 bags of *C. majus* seeds were prepared and buried in the soil at a depth of 5 cm at Scotia Seeds (56°69′99′′ N, 2°65′56′′ W; Angus, Scotland, UK). In each bag, 20 seeds were mixed with 20 g of the local soil and placed on a mesh tissue that was then folded and stapled. Maximum and minimum air temperatures were monitored every 3 days above the experiment at ground level. Seeds were sown on 11 September 2016. Fortnightly, a bag of seeds was recovered and sent to Wakehurst Place for TZ testing and embryo measurements, performed as described above. The last seed bag was retrieved on 2 March 2017.

### Storage behaviour

To test the effect of storage at different relative humidities, fresh seeds of *C. majus* were placed in airtight jars over solutions of lithium chloride (LiCl), generating 15%, 60% and 80% RH at 20 °C. The quantities of LiCl used per 1 L deionised water were 735 g, 300 g and 170 g, respectively (Gold & Hay 2014). Seeds were stored at the same temperature for 0 (control), 6 or 12 months; 110 seeds being used for each treatment. After each storage period, seeds were tested for germination at 5 °C as described above. The ten remaining seeds were stained in TZ solution and assessed for viability.

### Desiccation tolerance during embryo growth

The aim of this experiment was to test if and when *C. majus* seeds lose the ability to tolerate drying back after the start of embryo growth. Four dishes of 25 seeds and ten dishes of 15 seeds were sown on 9‐cm diameter Petri dishes containing 1% agar‐water and kept permanently at 5 °C as a control treatment. Other seeds were randomly sampled using a seed counter and sown, at 5 °C, on 1% agar‐water in four plastic boxes, each containing 450 seeds. Three, 28, 56 and 84 days after sowing, a box was retrieved from the incubator; all the seeds in it were blotted with paper to remove excess water; and split in two subsamples. Each subsample of 225 seeds was then placed in a desiccator jar above LiCl solution either at 15% or 60% RH as described above. Seed equilibrium RH (eRH) was monitored daily using a Rotronic hygrometer fitted with a HC2‐AW sensor and USB interface, connected to a laptop PC running HW4‐E software (Rotronic Instruments, Crawley, UK). When seeds reached eRH, they were rehydrated over water for 24 h, and sown on agar‐water for germination testing and embryo growth measurements.

### Statistical analysis

Statistical analysis of the results was performed using the software R 3.4.0 (R Core Team 2017). Embryo growth in the different experiments was tested by fitting linear models. In these models, cumulative E:E was the dependent variable, time a continuous independent variable, and the experimental treatments were the categorical independent variables. A common intercept was forced, representing the initial E:E ratio. Analysis of covariance (ancova) was used to compare the slope of each treatment (*i.e*. rate of embryo growth) against the control (5 °C in light without chemicals or pre‐treatment).

In all experiments, the effect of each treatment on the final germination percentages was tested by comparing it against the control (5 °C in light without chemicals or pre‐treatment) using Generalised Linear Models (binomial distribution, logit‐link). When a treatment resulted in no germination (see Results), the obvious difference meant that it was not included in the analysis.

The mean time for germination (MTG) was calculated as follows:

$$\sum (n^{*}t)/\sum n$$

where *n* was number of seeds germinated (radicle emergence) at each time *t*. The log10 of the value obtained was regressed against the probit of the final germination percentage obtained for each desiccation treatment to describe the change in seed vigour, following the approach of Powell *et al*. (1991).

## Results

Seeds of the 2015 seed lot had an initial moisture content of 17.8 ± 5.5% (± SE) and an average fresh mass of 1.89 ± 0.06 mg. They were assessed as 90% viable after TZ staining.

### Internal morphology

**Figure 1:**
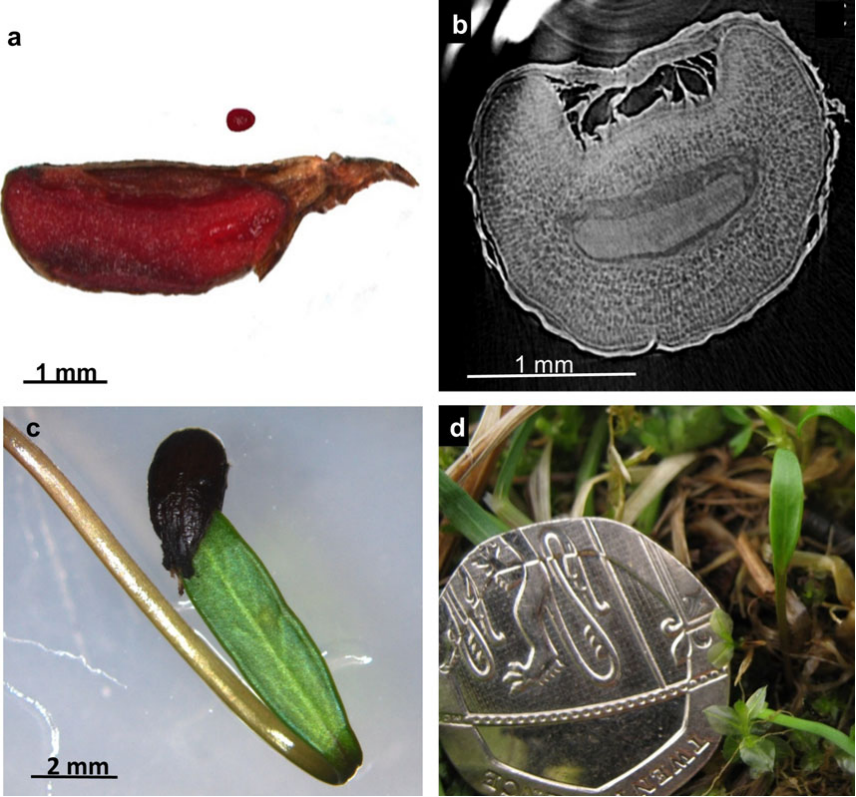
Embryo morphology in *Conopodium majus*. (a) Initial embryo size in a longitudinally dissected seed treated with tetrazolium chloride, with uniform red staining of endosperm and embryo indicating that the seed is viable. The embryo was excised from the seed to show its initial size. (b) Transverse mid‐section of *C. majus* seed obtained after 56 days at 5 °C during which time the embryo has grown within the seed. The central epicotyl portion of the embryo has differentiated but the tissue does not show any discontinuity, indicating this dicotyldonous species produces a pseudo‐monocotyledonous embryo. (c) Cotyledon emergence during seed germination. (d) Seedling *in situ*, at Dalraoch Farm, Scotland, in April 2016, 7 months after seed dispersal.

*Conopodium majus* embryos had a single entire‐sheath cotyledon (Fig. 1c, d). Using a stereomicroscope and observing whole excised embryos, no trace of a second cotyledon was visible at any stage of embryo growth and during germination (Fig. 1). The initial relative embryo size (E:E ratio) was 0.119 ± 0.005 and 0.125 ± 0.008 for seeds collected in 2015 and 2016, respectively. After 56 days of imbibition at 5 °C of the 2015 seed lot, E:E had increased to 0.344 ± 0.040. Even at this stage, there was no evidence of two cotyledons according to Micro‐CT imaging of the transverse section of the seed (Fig. 1b). Seeds germinated when embryos approached the endosperm length (*i.e*. E:E ratio was close to 1). Soon after germination, a bulbil formed by enlargement of a portion of the hypocotyl. No true leaves were produced until the second year after germination, and these appeared to develop from the bulbil.

### Embryo growth

**Figure 2:**
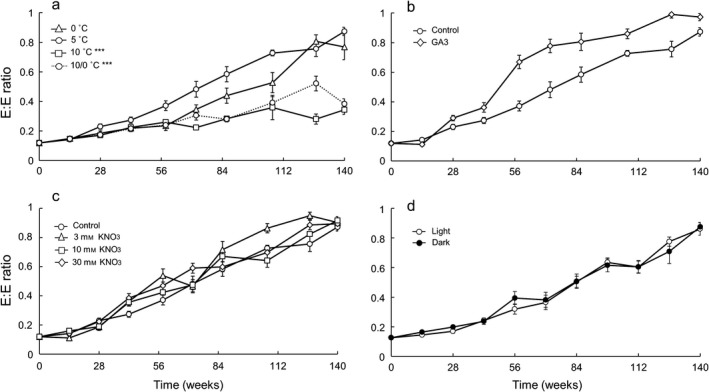
Increase in embryo to endosperm (E:E) ratio with time. Each point represents the average ± SE for the E:E ratio of ten living seeds. (a) Effect of temperature; (b) effect of externally applied GA_3_ on E:E at 5 °C; (c) effect of KNO_3_ concentration on E:E at 5 °C; (d) effect of light (12 h/12 h) and darkness on E:E at 5 °C. Differences in the rate of embryo growth were tested with ancova, whereas differences in final germination between control and treatments were tested using generalised linear models with logit‐link function. ***indicates significant (*P *< 0.001) differences between the treatments and 5 °C in (a).

There was a significant positive relationship between time and E:E at 5 °C (Student's *t* = 16.699, *P* < 0.001, slope = 0.0054), indicating that the embryo grew as time (in days) progressed. An increase in relative embryo size was already detected after 28 days from imbibition. The optimum temperatures for embryo growth were 0 and 5 °C, whereas the other temperatures resulted in slower growth rates (Fig. 2a). In particular, the slopes of the fitted regression lines were significantly lower at 10/0 °C (Student's *t* = −7.268, *P* < 0.001, slope = 0.0026) and 10 °C (Student's *t* = −9.094, *P* < 0.001, slope = 0.0019). The addition of GA_3_ and KNO_3_ (Fig. 2b, c) did not produce a significant difference in embryo growth rate (*P* > 0.05). Similarly, no significant differences (*P* > 0.05) were recorded in the rate of embryo growth between seeds exposed to light for 12 h day^−1^ and seeds incubated in constant darkness (Fig. 2d).

### Germination: radicle emergence

Germination started 12 weeks after sowing in the GA_3_ treatment, after 14 weeks when 3 mm KNO_3_ was present, and after 18 weeks in the control at 5 °C (without chemical treatment). MTG was calculated from the germination data for the temperature, GA_3_ and KNO_3_ treatments (Table 1). On average, germination in the control was recorded 20 days later than in the GA_3_ treatment. A significant difference was detected, from generalised linear models, when final germination was compared across treatments after 34 weeks of incubation. Only one seed incubated at 10 °C germinated, whereas the alternating temperature 10/0 °C significantly reduced final germination in comparison with the 5 °C control (25.2% *versus* 98.0%; Wald's z = −7.797, *P* < 0.001; Table 1). No significant difference was found between the other treatments and the control (Table 1).

**Table 1:** Effects of temperature and chemical additives on germination of *Conopodium majus* seeds. MTG is the average value (± SE) for the four replicates used in each test. General linear models were used to test the significance of responses compared to the control condition of 5 °C regarding final germination (G%). *P*‐values, *z* statistics and the lower and upper confidence intervals (CI) are reported

Treatment	MTG (days)	SE	G %	*P*‐value	z‐value	Lower CI	Upper CI
0 °C	148.85	3.03	88.75	0.126	−1.52	0.80	0.93
5 °C (control)	150.75	0.88	98	–	–	0.88	0.97
10 °C	238^a^	–	1.08	0.000	–	–	–
10/0 °C	213.24	2.03	25.20	0.000	−7.79	0.17	0.34
GA_3_	129.93	1.81	100	0.745	0.32	0.89	0.98
3 mm KNO_3_	141.59	1.28	96.95	0.722	0.35	0.89	0.98
10 mm KNO_3_	144.31	1.91	95.95	0.722	0.35	0.89	0.98
30 mm KNO_3_	156.84	1.44	92.68	0.362	−0.91	0.84	0.95

a Only one seed germinated in this treatment, after 238 days. As it was clearly different from the control, the 10 °C treatment was not tested with GLM.

**Figure 3:**
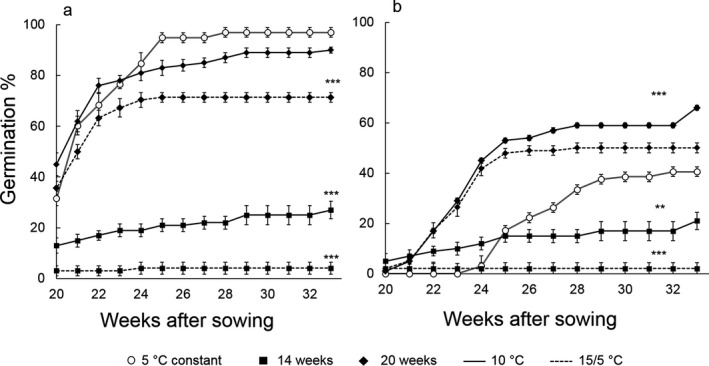
Germination (a) and cotyledon emergence (b) after three different cold stratification treatments (14, 20, 33 weeks at 5 °C). Seeds were maintained at 5 °C for 14 weeks (closed squares) or 20 weeks (closed diamonds) followed by transfer to two warmer temperatures: 10 °C (solid line) or 15/5 °C (dashed line). Differences in final germination between the control (seeds held at 5 °C for the full 33 weeks of the experiment) and treatments were tested using generalised mixed models with logit‐link function. ***(*P *< 0.001) and **(*P *< 0.01) signify treatments that are significantly different from the control (5 °C, open circles). Cold stratification for 0 weeks (*i.e*. germination temperatures of 10 and 15/5 °C) and for 6 weeks resulted in no germination.

In the cold stratification experiment, no germination occurred in the treatments that were incubated at 10 °C and 15/5 °C without previous exposure to cold. Germination started during cold stratification at 5 °C in the light. Therefore, some seeds had already germinated when they were moved to 10 °C and 15/5 °C, both after 14 and 20 weeks of stratification (Fig. 3a). Final germination, scored after 33 weeks from the beginning of the experiment, was higher in the 5 °C control (96.9 ± 1.9%) and significantly (Wald's z ≤ −3.930, *P* < 0.001) lower in all other treatments, except when 20 weeks of stratification were followed by germination at 10 °C (90.0 ± 1.2%; Wald's z = −1.280, *P* = 0.201). However, the increment in germination from the first scoring (week 20) to the end of the experiment was higher for seeds in the 5 °C constant control (+ 65.3%) than for seeds that were moved to 10 °C (+ 45.0%) and 15/5 °C (+ 35.7%) after 20 weeks at 5 °C (Fig. 3a).

### Germination: cotyledon emergence

Some cotyledon emergence also occurred at 5 °C, although less than for radicle emergence (Fig. 3b). Final cotyledon emergence was significantly (Wald's z = 3.512, *P* < 0.001) higher for seeds that were moved to 10 °C after 20 weeks at 5 °C, compared with the cold control; reaching 66.0 ± 9.0% (moved to 10 °C) compared to 40.5 ± 7.0% for the control (constantly at 5 °C). Seeds that were incubated for 14 weeks at 5 °C and then moved to 10 °C had a shorter gap between germination and cotyledon emergence, compared to seeds of the 5 °C control, but with a significantly (Wald's z = −2.978, *P* = 0.002) lower final cotyledon emergence (21.0 ± 2.5%; Fig. 3b).

### Embryo growth in the soil

**Figure 4:**
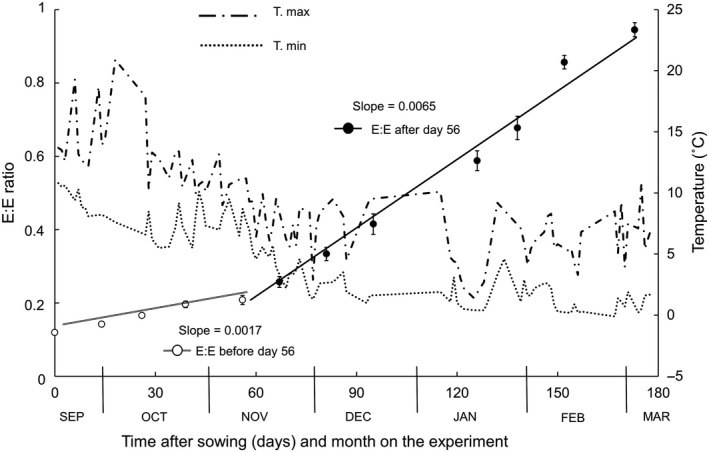
Embryo development in seeds buried in the ground at Dalraoch Farm, Scotland, in relation to recorded site air temperatures (maxima and minima). Data represent average (±SE) E:E ratio for 20 seeds at each sample time. The slopes for dependency of E:E in time were estimated by linear fits to the first five measurements (first 56 days; open circles) and the following >100 days (closed circles) to highlight the differences in rate of embryo growth as minimum temperatures dropped regularly below 5 °C.

As in the laboratory experiment, embryo growth in the field was detected within 284 days from sowing. In the period after dispersal, the rate of internal embryo growth was 0.0017 day^−1^. In late November, 10 weeks from sowing, the average minimum temperatures dropped constantly below 5 °C and the rate of embryo growth accelerated to 0.0065 day^−1^. Thus, embryo growth was 3.82‐fold faster when temperatures dropped below 5 °C, a significant increase from the previous period (Student's *t* = 6.685, *P* > 0.001; Fig. 4). In the last retrieved samples, 173 days after the beginning of the experiment, *in situ* germination was apparent, with half the seeds having emerged radicles. At this stage, the average E:E of the whole sample including germinated seeds was 0.945 ± 0.019.

### Storage behaviour

**Figure 5:**
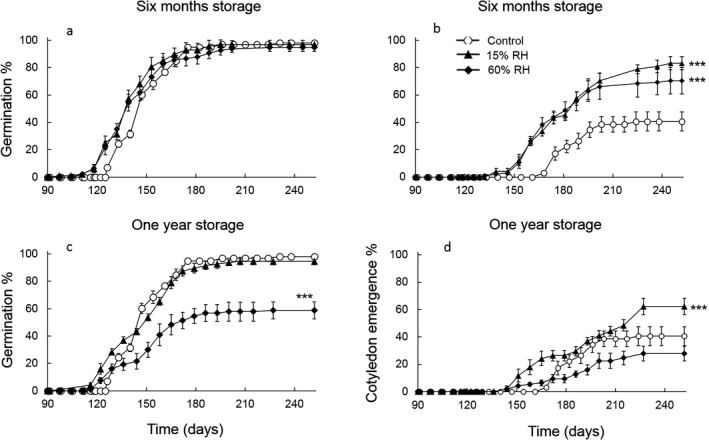
Germination and cotyledon emergence progress curves at 5 °C after seed storage at room temperature (ca. 20 °C) and either 15% RH (dry room treatment) or 60% RH (over lithium chloride solution) for 6 months (a, b) or 1 year (c, d). Germination data are shown from 90 days onwards. The control treatment (open circles) consisted of seeds sown fresh after collection. Differences in final germination or cotyledon emergence between the control and the treatments were tested using generalised mixed models with logit‐link function. ***P < 0.001, signify treatments that are significantly different from the control. Each treatment consisted of four replicates of 25 seeds.

At 5 °C, final germination percentages of seeds stored for 6 months (96.9 ± 1.9%) or 1 year (95.8 ± 3.1%) at 15% RH and for 6 months at 60% RH (94.7 ± 3.1%) did not differ significantly from fresh seeds (98.0 ± 2.0%). Final germination of seeds held at 60% RH for 1 year (60.8 ± 4.7%) was significantly lower than the control (Wald's z = −4.930, *P* < 0.001). TZ viability (70%) of these seeds was also lower than the control (90%); whereas all other treatments had >80% viable seeds. No germination occurred in seeds stored at 80% RH for any period, with 0% viable seeds (Fig. 5a, c). Storage also affected the onset of germination. Germination began 29, 10, 15 and 4 days earlier than the control in the 6 months 15% RH, 1 year 15% RH, 6 months 60% RH and 1 year 60% RH treatments, respectively (Fig. 5a, c). An advance in cotyledon emergence was also noted in stored seeds, occurring on average 27 days earlier for seeds stored for 6 months at 60% RH and 1 year at 15% and 60% RH (Fig. 5b, d). However, the proportion of seeds able to complete cotyledon emergence was less than the seeds able to germinate (radicle emergence) for all treatments, but the decrease was more pronounced for seeds that were fresh or stored for 1 year (Fig. 5). Cotyledon emergences were significantly higher (Wald's z ≥ 0.300, *P* < 0.001) than the control (40.5 ± 7.0%) for seeds stored for 6 months at 15% (83.1 ± 4.0%) and 60% RH (70.3 ± 5.1%) and for seeds stored for 1 year at 15% RH (62.1 ± 8.1%). The reduction in cotyledon emergence of seeds stored for 1 year at 60% RH (27.8 ± 5.0%) was consistent with their lower germination but the difference from the control was not significant (Wald's z = 0.306, *P* = 0.080).

### Desiccation tolerance during embryo growth

**Figure 6:**
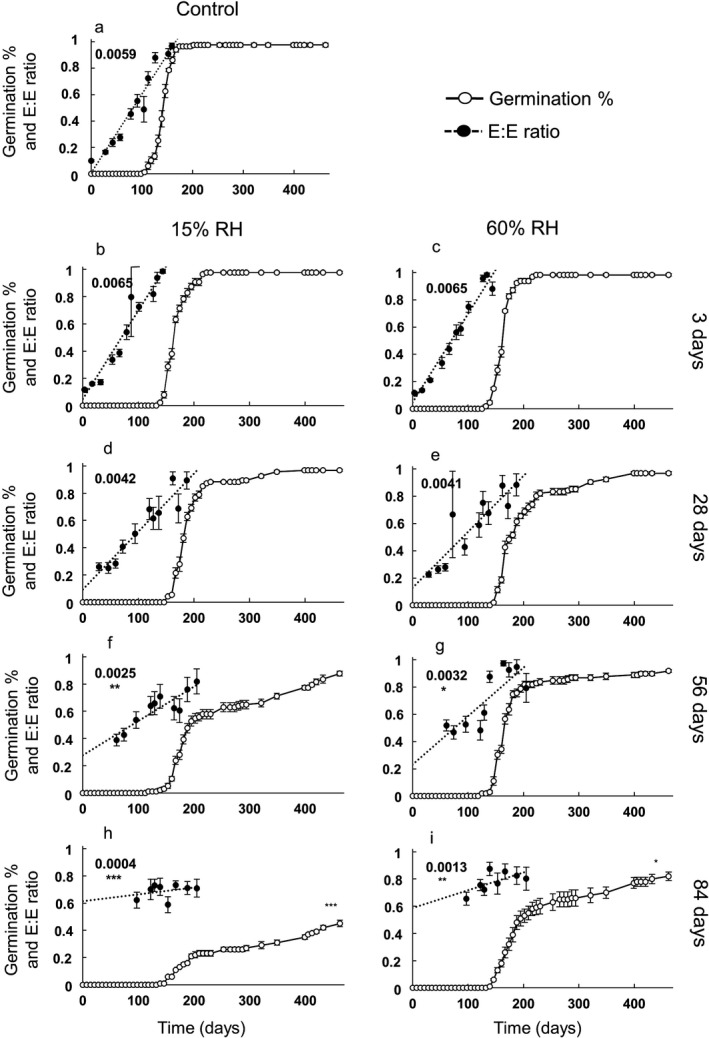
Effects of embryo growth at 5 °C on desiccation tolerance (drying back) in *Conopodium majus* seeds. E:E ratio (closed circles) and germination (radicle emergence; open circles, expressed as a proportion on the total of viable seeds) progress curves at 5 °C (a) after seeds were pre‐treated at 5 °C for 3, 28, 56 and 84 days followed by drying back to 15% RH (b, d, f, h) and 60% RH (c, e, g, i). E:E ratios are based on ten living seeds ± SE. The control treatment (a) consisted of seeds to which no desiccation treatments were applied. Differences in final germination between control and treatments were tested using generalised mixed models with logit‐link function. Regression lines (dotted lines) were fitted to the embryo growth progression (change in E:E ratio per day) and the difference between the slopes of the control and of the treatments (indicated in each chart) tested with an ancova. ***P < 0.001, **P < 0.01 and *P < 0.05 signify significant differences between the treatment and the control.

In the control (5 °C), visible germination was evident 98 days after sowing, when the average E:E was close to 0.6, while seeds after 91 days incubation had an E:E of 0.553 ± 0.047 (Fig. 6a). Therefore, the last dehydration treatment (84 days after sowing) occurred when embryos had grown to over half the endosperm length. Subsequent embryo growth rates did not differ significantly from the control in seeds that were dehydrated 3 and 28 days after imbibition, indicating no loss in vigour (Fig. 6b–e). Later dehydration events (56 and 84 days post‐imbibition) significantly reduced the embryo growth rate (Fig. 6f–i). In particular, when seeds were dehydrated to 15% RH after 84 days of cold imbibition, the growth rate (*i.e*. E:E change in time) fell from 0.0059 day^−1^ in the control to 0.0004 day^−1^ (Student's *t* = −3.452, *P* < 0.001). A consistent, but less pronounced, decrease in the rate of embryo growth was observed in seeds dehydrated to 15% RH after 56 days (rate = 0.0025 day^−1^; Student's *t* = −2.878, *P* = 0.005), and in seeds dehydrated to 60% RH after 56 (rate = 0.0032 day^−1^; Student's *t* = −2.249, *P* = 0.027) and 84 (rate = 0.0013 day^−1^; Student's *t* = −2.941, *P* = 0.004) days.

**Figure 7:**
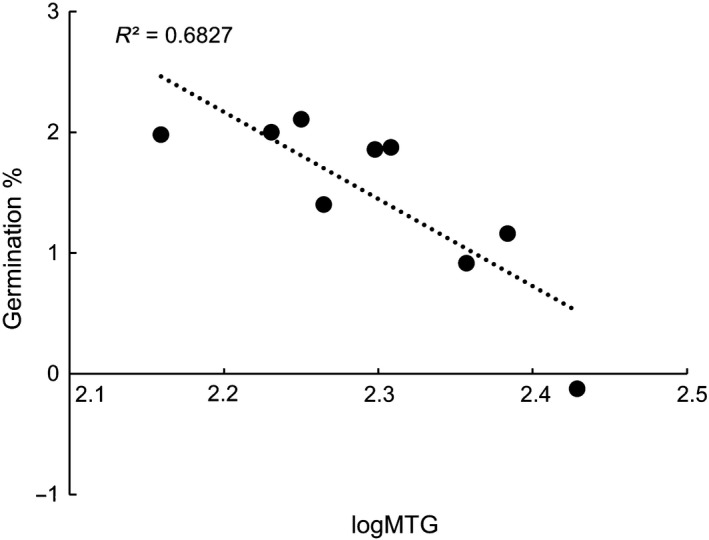
Negative relationship between mean time to germination (MTG, plotted on a log scale) and final germination (% of radicle emergence plotted on a probit scale) in seeds exposed to desiccation during embryo development (both treatments). A probit value = 0 correspond to 50% final germination.

Desiccation treatments had a significant effect also on final germination, scored 462 days from the beginning of the experiment. Radicle emergence was significantly lower for seeds dehydrated to 15% (45.0 ± 2.3%; Wald's z = −6.042, *P* < 0.001) and 60% RH (82.0 ± 3.0%; Wald's z = −2.491, *P* = 0.012) after 84 days of embryo growth compared with the control (97.6 ± 1.5%; Fig. 6f, g). The proportion of fresh but non‐germinated seeds was higher in the seeds desiccated after 84 days of embryo growth compared with the other treatments, whereas the proportion of non‐viable seeds did not increase significantly. The regression of MTG against the probit of the final germination percentage of all the treatments resulted in a negative relationship (R^2^ = 0.68) in which longer MTG corresponded with lower final germination over the time‐course of the experiment (Fig. 7).

## Discussion

The findings of this study confirm that seeds of *C. majus* have pseudomonocotyledony, morphological dormancy and a relatively high tolerance to desiccation. In reviewing pseudomonocotyly, Haines (1979) proposed that the origin of a single cotyledon in some *Apiaceae* species is a consequence of their adaptation as geophytes. The necessity to push the radicle and the hypocotyl deep into the soil may have led to the evolution of a cotyledonary tube, originated by the fusion of the cotyledon petioles rather than the suppression (abortion) of one of them (Haines 1979). To date, the only detailed anatomical description of embryo and seedling development in pseudomonocotyledoneous *Apiaceae* is related to the Asian genus *Acronema* (Kljuykov *et al*. 2014). No details had been provided previously on the growth of the single cotyledon inside the seed. In a ripe seed of *C. majus* the embryo has an average initial size of 0.18 mm and differentiation into a radicle and cotyledon is not yet clear. While in seeds cold‐stratified for 56 days the radicle and cotyledon are visually distinct, there is still no clear subdivision into two cotyledonary sheaths. The fusion of the two cotyledons is even more evident in the young seedlings (Fig. 1c, d), supporting Haines’ (1979) interpretation that pseudomonocotyledony might serve to assist young plants of geophytes to penetrate into the soil.

Embryo growth and germination (radicle emergence) in *C. majus* are inhibited by temperatures warmer than 10 °C and only promoted by a narrow range of low temperatures, between 0 and 5 °C. Based on both laboratory and field studies, embryo growth starts shortly after imbibition at the optimal temperatures. In the field, the time required to reach 50% germination can extend to 6 months, from dispersal in late summer/autumn to germination in early spring. However, embryo growth is much faster during the winter months, during colder temperatures, indicating precise thermal control of this process. As embryo growth inside the seeds transitions seamlessly into germination (radicle emergence) at the same temperature, the species should be classified as morphologically dormant (MD) rather than morphophysiologically dormant (MPD), as there is no evidence of a physiological block to embryo growth (Baskin & Baskin 2004). The minimal response to GA_3_ and nitrate is consistent with the lack of MPD. Similarly, embryos of the woodland indicator species *Anemone nemorosa* L. (*Ranunculaceae*) start to grow immediately after dispersal, although warm stratification improves the response (Mondoni *et al*. 2008). Other woodland species, including *Corydalis solida* (L.) Clairv. (*Papaveraceae*)*, Narcissus pseudonarcissus* L. (*Amaryllidaceae*) and *Scilla bifolia* L. (*Asparagaceae*), have been classified as MPD, and their embryos start to grow when the temperatures are still relatively high (Vandelook & Van Assche 2008a, 2009; Newton *et al*. 2013, 2015). Among other woodland species from the *Apiaceae* family that do not require warm stratification for embryo growth, *Sanicula europaea* L. responds like *C. majus*, requiring a low (5 °C) optimal temperature for embryo growth and germination, and showing the lack of developmental arrest between the two growth phases (Vandelook & Van Assche 2008b). In contrast, *Aegopodium podagraria* L. and *Chaerophyllum temulum* L. (*Apiaceae*) require cold temperatures for embryo growth, but can then germinate over a wider range of temperatures once the internal growth of the embryo within the seed is completed (Vandelook *et al*. 2007, 2009). A slow germination rate at cold temperatures has an ecological benefit in stable and predictable habitats, such as temperate deciduous forests, where suitable conditions for imbibition and embryo growth occur throughout the winter (Vandelook *et al*. 2012). In the *Apiaceae*, these germination requirements and the relatively small initial embryo size are traits associated with a preference for stable and shady habitats, whereas species from open habitats tend to have higher relative embryo sizes (Vandelook *et al*. 2012).

Embryo growth in *C. majus* is indifferent to light, which can be indicative of the ability of the seeds to germinate when completely covered by soil or leaf litter. The need of light for germination, in temperate forest understorey herbs, is typical of species with seed mass < 1.5 mg (Jankowska‐Błaszczuk & Daws 2007), and *C. majus* seeds are relatively large in comparison (1.9 mg). In forest habitats, light indifference has been correlated with the ability to quickly colonise gaps in the forest canopy (Pearson *et al*. 2002), whereas in *C. majus* this response can be regarded as an adaptation to its geophytic life form. Its seeds can germinate even at a depth of 3–5 cm, which is ideal for the development of the young tuber, whereas the epicotyl grows towards the light. On the other hand, embryo growth and germination were significantly higher when seeds were exposed to a constant temperature regime of 0 or 5 °C rather than at temperatures fluctuating between 10 °C during the day and 0 °C at night. It seems that 10 °C is *supra*‐optimal for embryo growth in this species and slowed down the process that should have progressed at 0 °C. The detection of fluctuating temperatures is a gap‐sensing mechanism, and some species have a threshold of daily temperature fluctuation that can trigger their germination (Pearson *et al*. 2002). In contrast, species adapted to germinate in low light conditions and that possess big seeds can also germinate in the absence of fluctuating temperatures (Thompson & Grime 1983). In the case of *C. majus*, another factor to consider is that embryo growth takes place in the coldest months of the year, when the span of daily temperature fluctuation is lower. Taken together, the indifference to light and alternating temperatures indicate a preference for germination under a protective layer of leaf litter or snow, an environmental preference also found in sub‐Mediterranean mountain geophytes (Fernández‐Pascual *et al*. 2017).

Although seedlings eventually emerge at 5 °C, the temperatures that promote embryo growth and radicle emergence do not fully correspond with the temperatures that promote cotyledon emergence, the latter preferring slightly higher temperature for faster and higher emergence. Presumably, the thermal control of different growth phases varies, as shown for embryo growth and radicle emergence in *Aquilegia barbaricina* Arrigoni & E.Nardi (*Ranunculaceae*; Porceddu *et al*. 2017). In *Heracleum spondylium* L. (*Apiaceae*), cold temperatures promote embryo growth by stimulating the conversion of the proteins stored in the endosperm into soluble nitrogen compounds, promoting radicle emergence (Stokes 1953). Although no seedlings were scored during the field germination experiment, cotyledon emergence in the natural environment was observed in late March/early April, when the temperature rises, the canopy is still open and few herbaceous understorey species have emerged. Overall, the phenology of germination and seedling emergence in *C. majus* is comparable to other woodland specialists whose germination is finely synchronised with the seasonal environmental changes in temperate forests (Mondoni *et al*. 2008; Vandelook & Van Assche 2008b; Newton *et al*. 2013, 2015). Thus, *C. majus* can be regarded as a spring ephemeral species that reproduces between January and May.

Fresh seeds of *C. majus* tolerate desiccation to 15% RH and do not lose viability for at least 1 year if stored dry at 20 °C. However, if stored at higher humidity (60% or 80% RH), seeds lose from half to all viability in 6 months to 1 year. This indicates the need for the native seed industry to store seeds of this species under dry conditions. Storage increases cotyledon emergence in some cases, and further research is needed to clarify the physiological effects of dry storage on seedling performance. Once *C. majus* embryos attain a certain stage of development, reaching five times their initial size, they retain a high level of desiccation tolerance, albeit accompanied by slower subsequent seed germination, as determined by MTG. Based on insights gained in this study, germination in the natural environment appears likely within the first winter and spring period, with few seeds becoming incorporated into the soil seed bank. This supports an earlier observation that *C. majus* seeds do not form a persistent soil seed bank (Thompson *et al*. 1997). Nonetheless, germination of *C. majus* seeds is so protracted that not all seeds in a cohort might achieve cotyledon emergence and establishment before the following summer. In this case, it is important to know if seeds with a grown embryo remain tolerant to dehydration. Although seeds with morphological dormancy can persist in the soil for some years (Hawkins *et al*. 2007), a decrease in desiccation tolerance as the embryo develops is common. As with *C. majus*,* Panax ginseng* C.A.Mey. (*Araliaceae*) seeds exhibit some sensitivity after drying back at different stages of embryo growth (0.10, 0.46 and 0.91 E:E; Han *et al*. 2016). Similarly, in a study of the germination ecology of *Lomatium dissectum* (Nutt.) Mathias & Constance (*Apiaceae*; Scholten *et al*. 2009), a species subjected to seasonal habitat drought, seed drying after a period of imbibed cold stratification decreased viability by 30%, and the surviving seeds showed a reduced germination rate (vigour). Although such desiccating conditions are not likely to occur in most of the European Atlantic distribution of *C. majus*, mountain populations at the southern boundary of the distribution range can experience a continental Mediterranean climate. In this context, the capacity to survive desiccation stress, even if at the cost of a slower embryo growth rate and thus delayed germination, may have allowed the survival of *C. majus* in those more unpredictable habitats at the southern margin of its distribution.

In conclusion, reproduction by seed in *C. majus* follows a strategy shared by geophytes adapted to deciduous temperate forests. The small embryo is capable of slow growth within the seed at low temperatures so that germination is timed for early spring, whereas cotyledon growth occurs later at warmer temperatures. Embryo growth and germination (at least radicle emergence) appear to be consecutive processes regulated by the same thermal control, with no role for light or alternating temperature or a requirement for dormancy‐breaking chemicals. Seeds of this species have morphological and germination traits that suit its broad ecological niche in Europe, potentially including drier sites as embryo growth over winter does not change seed desiccation tolerance profoundly. The evolution of fused cotyledons likely enables the radicle and the hypocotyl to be pushed deep into the soil where a tuber can develop. Further studies of populations growing in different habitats and across the latitudinal distribution range of the species may provide insights on the role of embryo traits in shaping the functional biogeography of this species.
